# A Remarkable Accident

**Published:** 1898-03

**Authors:** J. Fitz-Mathew

**Affiliations:** West Sound, Washington


### A REMARKABLE ACCIDENT.

J. Fitz-Mathew, M. D., West Sound, Washington.

In a saw-mill a rapidly revolving shaft passed along a slit in the flooring over the basement, having a space of six inches on either side of the shaft, the flooring being of one and a half inches in thickness. A young woman, two months pregnant—*primipara*—had been repeatedly cautioned not to step over this shaft in visiting her husband, working in the mill. One day, in stepping over it, her dress was caught in a coupling-nut, and in a few seconds she was safely delivered in the basement, *sans* clothing, excepting the stockings, and with only a few abrasions of the skin. It was found that a couple of inches of the flooring on one side was broken away. I can vouch for the accuracy of these details.

This case is of interest to obstetricians, constituting a "breech presentation" of novel character, in which the transverse diameter was, fortunately, arranged parallel to the revolving shaft, and the anterio-posterior—a small, slight figure—found a way of exit by the edge of the flooring giving way.

Now, it is evident that the hem of the skirt behind was caught by the "nut" on the shaft, the dress rapidly wound down, drawing the body down, doubled up like a jack-knife, and a wad of clothing under the breech prevented any serious damage to it in its forced expulsion. This young woman was safely delivered at term.

A fine lad, aged twelve years, who endured his sufferings with remarkable fortitude, while walking along the seashore through a mass of rocks, saw a rock of some half-ton in weight become detached from the side of the cliff and rolling down on him. He made an effort to escape by springing up the face of a large rock on the seaward side, but was too late. I was in attendance upon him some sixteen hours after the accident. Under ether I found that an elongated corner of the revolving rock had entered the pelvic cavity, tearing away the levator ani, and stripping the coccyx, on one side, of all its attachments. Passed catheter and found bladder uninjured. It was a most marvelous escape for him, as will be readily seen. Coccyx was not fractured. In the treatment of this case, Ars. 6c to 2c, with sol. of pernang. potash, corrected very promptly a very fetid discharge with greenish hue from the lacerated tissues. Swabbing with fluid extract of Calendula and packing with sublimated cotton was the dressing. After considerable trouble with the rectum for a while from inertia, the lad was perfectly well in less than two months.

